# Evaluating the Impact of Preoperative Nutritional Status on Surgical Outcomes and Public Health Implications in General Surgery Patients

**DOI:** 10.7759/cureus.76633

**Published:** 2024-12-30

**Authors:** Rabail Bashir Keerio, Muhammad Ali, Kamran A Shah, Atif Iqbal, Asif Mehmood, Sikandar Iqbal

**Affiliations:** 1 General Surgery, Russell’s Hall Hospital, Dudley, GBR; 2 Surgical Unit, Allama Iqbal Teaching Hospital, Dera Ghazi Khan, PAK; 3 General Surgery, University Hospitals Birmingham, Birmingham, GBR; 4 Consultant Surgery, Jinnah International Hospital, Abbottabad, PAK; 5 General Surgery, Bannu Medical College, Bannu, PAK; 6 General Surgery, Institute DHQ Hospital Dassu, Upper Kohistan, PAK

**Keywords:** infection, malnutrition, postoperative complications, preoperative nutritional status, surgical outcomes

## Abstract

Background: Malnutrition is linked to more postoperative problems, a longer recovery period, and a higher death rate; preoperative nutritional condition is a critical factor in surgical outcomes.

Objective: This study aimed to evaluate the impact of preoperative nutritional status on surgical outcomes in general surgery patients and explore its broader public health implications.

Methodology: A two-year observational research with 440 adult patients undergoing general surgery was carried out between January 2022 and December 2023. Based on preoperative nutritional tests, such as Nutritional Risk Screening (NRS-2002), Body Mass Index (BMI), and blood albumin levels, participants were divided into two groups: nutritionally sufficient and nutritionally compromised. Postoperative problems, such as infections, wound healing, duration of hospital stay, readmissions, and 30-day mortality, were examined in the data.

Results: Nutritionally compromised patients exhibited significantly worse outcomes, including higher rates of postoperative infections (80 out of 220, 36.36% vs. 30 out of 220, 13.64%), delayed wound healing (50 out of 220, 22.73% vs. 20 out of 220, 9.09%), longer hospital stays (9.87 ± 3.58 vs. 6.53 ± 2.31 days), increased readmission rates (40 out of 220, 18.18% vs. 10 out of 220, 4.55%), and higher 30-day mortality (25 out of 220, 11.36% vs. 5 out of 220, 2.27%) compared to the nutritionally adequate group. Long-term follow-up showed persistent differences in infection rates and wound healing, supporting the prolonged impact of poor nutritional status.

Conclusion: Preoperative malnutrition significantly affects surgical outcomes, emphasizing the importance of nutritional optimization in preoperative care to enhance recovery and minimize complications.

## Introduction

The importance of preoperative nutritional status in predicting surgical outcomes in a variety of patient groups is becoming more acknowledged [1,2]. Overt or subclinical malnutrition has been repeatedly linked to greater incidence of surgical complications, longer hospital stays, increased morbidity, and even death [3]. The relationship between nutritional health and surgical results requires special consideration in the context of general surgery, where patient profiles and procedures vary greatly. Optimizing both individual patient treatment and a more general healthcare strategy requires an understanding of this link [4].

The complex processes that underlie the connection between healing and nutrition depend on how the body reacts metabolically to surgical stress [5]. Inflammatory reactions are aggravated, wound healing is delayed, and immune function is compromised by poor nutritional condition [6]. On the other hand, individuals who have sufficient nutritional reserves are more resilient to the physiological demands of surgery, recuperate more quickly, and have fewer problems [7]. Almost 40% of general surgery patients are still at risk of malnutrition despite improvements in perioperative care and surgical methods, sometimes as a result of undiagnosed nutritional deficiencies or underlying chronic conditions [8].

Beyond individual results, these findings have public health implications that impact healthcare systems and policy [9]. Longer hospital stays and readmissions result in increased healthcare expenses for nutritionally challenged patients, which adds to the financial strain on hospitals and national health systems [10,11]. It may be possible to address these systemic issues and enhance surgical outcomes by including nutritional evaluation and optimization into preoperative treatment routes [12].

Although preoperative malnutrition has been shown to have advantages, there are still gaps in our knowledge of how much of an influence it has in various surgical scenarios. This emphasizes the need for thorough studies that take into account wider public health consequences in addition to assessing the results for specific patients. This study evaluated the impact of preoperative nutritional status on surgical outcomes in general surgery patients, focusing on its association with postoperative complications and broader public health implications.

## Materials and methods

Study design and setting

This two-year observational study, which took place at Khalifa Gul Nawaz Teaching Hospital, Bannu and Allama Iqbal Teaching Hospital Dera Ghazi Khan in the Department of General Surgery, involved 440 adult patients undergoing general surgery. Based on preoperative evaluations, the patients were split into two groups: those who were nutritionally adequate (n = 220) and those who were nutritionally compromised (n = 220).

Inclusion and exclusion criteria

Adult patients having elective or emergency general surgery who were at least 18 years old, had undergone comprehensive preoperative nutritional evaluations, and gave their informed permission were included in the research. Patients who were pregnant or had fatal conditions unrelated to surgery, had insufficient medical records or dietary data, or were receiving minor outpatient treatments were not included.

Sample size

The total sample size for the study was 440 patients. The sample size was determined using power analysis to ensure 80% statistical power at a 5% significance level (α = 0.05). A presumed medium effect size (Cohen’s d = 0.5), commonly observed in studies examining the impact of preoperative nutritional status on surgical outcomes, was used for the calculation. Previous studies (Weimann et al. [13], Loon et al. [14]) have demonstrated medium effect sizes when comparing surgical complications, length of hospital stay, and postoperative recovery between nutritionally adequate and compromised groups [13,14]. The calculated minimum sample size required for adequate power was 384 patients, but to account for potential patient attrition and ensure robust results, the final sample size was adjusted to 440 participants. This adjustment also allowed for subgroup analyses and ensured the study had sufficient statistical power to detect meaningful differences in surgical outcomes.

The calculation was based on a significance level (α) of 0.05 and a power (1-β) of 80%, which are commonly used thresholds to detect clinically significant differences. In line with previous literature, a 10% dropout rate was estimated to ensure that the final sample size would be sufficient despite potential losses during follow-up. By including a larger sample, the study aimed to minimize biases such as selection bias and ensure the findings are generalizable to the broader population of general surgery patients [13,14].

Data collection

Clinical and demographic data (age, gender, comorbidities, type of surgery), preoperative nutritional evaluations (Nutritional Risk Screening [NRS-2002], Body Mass Index (BMI), serum albumin levels, and dietary history), and surgical outcomes (postoperative complications like infections, delayed wound healing, length of hospital stay, readmission rates, and 30-day postoperative mortality) were all gathered using a structured pro forma. Follow-up was carried out at three, six, 12, 18, and 24 months after surgery to document both immediate and long-term postoperative results, and patient records were checked for correctness.

Additional nutritional markers, such as prealbumin and transferrin, were not included in this study. However, these markers were excluded based on practical considerations, including the availability of resources and prior studies [15] that have demonstrated serum albumin as a reliable marker for nutritional status in surgical patients.

Statistical analysis

SPSS Version 25.0 (IBM Corp., Armonk, NY, USA) was used to analyze the data. Frequencies and percentages were used to convey categorical data, while mean ± standard deviation was used to summarize continuous variables. Multivariate analyses were performed to adjust for confounders such as age, gender, and comorbidities, which could influence surgical outcomes. Independent t-tests for continuous variables and chi-square tests for categorical variables were used to examine the relationship between nutritional status and surgical outcomes.

Minimizing bias and ensuring robustness in study design

To minimize bias and ensure the robustness of our findings, several steps were taken during the study design and analysis. First, we conducted a comprehensive preoperative nutritional evaluation using validated tools such as the Nutritional Risk Screening (NRS-2002) and serum albumin levels. To account for potential confounders such as age, gender, and comorbidities, we performed multivariate analyses, adjusting for these variables in our statistical models. This adjustment allowed us to isolate the effect of preoperative nutritional status on surgical outcomes, reducing the potential influence of confounding factors. Additionally, we ensured a large sample size (440 patients), which provided sufficient statistical power (80%) to detect meaningful differences in outcomes while accounting for potential patient attrition. A 10% dropout rate was anticipated, and the sample size was adjusted accordingly to ensure the study maintained sufficient power for subgroup analyses. By incorporating these steps, we aimed to minimize selection bias, confounding, and other biases, thus strengthening the validity and generalizability of our results.

Ethical approval

The Institutional Review Board and Ethical Committee of Bannu Medical College granted ethical approval (Approval No. 55/DiR&MJ/BMC/2024). Prior to data collection, all participants gave written informed permission, and patient confidentiality was maintained for the entire investigation.

## Results

The clinical features and demographics of individuals by nutritional status are shown in Table 1. Participants in the nutritionally compromised group were slightly older (55.11 ± 13.02 years) compared to those in the nutritionally adequate group (52.19 ± 11.82 years). The proportion of male participants was higher in the compromised group (125 (56.82%)) compared to the adequate group (115 (52.27%)). Comorbid conditions were more prevalent in the nutritionally compromised group, with higher rates of hypertension (70 (31.82%) vs. 50 (22.73%)), diabetes mellitus (50 (22.73%) vs. 30 (13.64%)), and cardiovascular disease (25 (11.36%) vs. 15 (6.82%)). Elective surgeries were more frequent in the compromised group (163 (74.09%)) compared to the adequate group (150 (68.18%)), while emergency procedures were less common in the compromised group (57 (25.91%)) compared to the adequate group (70 (31.82%)).

**Table 1:** Demographics and Clinical Characteristics of Study Participants by Nutritional Status Data are presented as n (%)

Characteristic		Nutritionally Adequate (n = 220)	Nutritionally Compromised (n = 220)
Age in Years	Mean ± SD	52.19 ± 11.82	55.11 ± 13.02
Gender	Male	115 (52.27%)	125 (56.82%)
	Female	105 (47.73%)	95 (43.18%)
Comorbidities	Hypertension	50 (22.73%)	70 (31.82%)
	Diabetes Mellitus	30 (13.64%)	50 (22.73%)
	Cardiovascular Disease	15 (6.82%)	25 (11.36%)
Type of Surgery	Elective	150 (68.18%)	163 (74.09%)
	Emergency	70 (31.82%)	57 (25.91%)

In comparison to the nutritionally adequate group, the nutritionally compromised group demonstrated significantly worse outcomes across all parameters (Table 2). A higher proportion of individuals in the compromised group were categorized as high risk based on Nutritional Risk Screening (NRS-2002) (80 (36.36%) vs. 20 (9.09%)). Compromised patients also exhibited lower albumin levels (<3.5 g/dL: 80 (36.36%) vs. 20 (9.09%)) and higher rates of being underweight (41 (18.63%) vs. 10 (4.55%)). Postoperative complications were more frequent in the compromised group, including infections (80 (36.36%) vs. 30 (13.64%)) and delayed wound healing (50 (22.73%) vs. 20 (9.09%)). These patients also had longer hospital stays (9.87 ± 3.58 days vs. 6.53 ± 2.31 days), higher readmission rates (40 (18.18%) vs. 10 (4.55%)), and higher 30-day postoperative mortality (25 (11.36%) vs. 5 (2.27%)). Overall, 130 (59.09%) of compromised individuals experienced postoperative complications compared to 50 (22.73%) in the adequate group, emphasizing the critical role of preoperative nutritional status in influencing surgical outcomes.

**Table 2:** Preoperative Nutritional Assessments and Surgical Outcomes by Nutritional Status Data are presented as n (%)

Characteristic		Nutritionally Adequate (n = 220)	Nutritionally Compromised (n = 220)
Nutritional Risk Screening- NRS 2002	Low Risk	150 (68.18%)	50 (22.73%)
	Moderate Risk	50 (22.73%)	90 (40.91%)
	High Risk	20 (9.09%)	80 (36.36%)
Body Mass Index	Underweight	10 (4.55%)	41 (18.63%)
	Normal Weight	130 (59.09%)	89 (40.45%)
	Overweight	60 (27.27%)	52 (23.63%)
	Obese	20 (9.09%)	38 (17.27%)
Albumin Levels	Low (< 3.5 g/dL)	20 (9.09%)	80 (36.36%)
	Normal (3.5–5.0 g/dL)	180 (81.82%)	117 (53.18%)
	High (> 5.0 g/dL)	20 (9.09%)	23 (10.45%)
Surgical Outcomes	Postoperative Complications	50 (22.73%)	130 (59.09%)
	Infections	30 (13.64%)	80 (36.36%)
	Delayed Wound Healing	20 (9.09%)	50 (22.73%)
	Length of Hospital Stay (Days)	6.53 ± 2.31	9.87 ± 3.58
	Readmission Rate	10 (4.55%)	40 (18.18%)
	30-Day Postoperative Mortality	5 (2.27%)	25 (11.36%)

Infection rates and delayed wound healing remained consistently higher in the nutritionally compromised group compared to the nutritionally adequate group throughout the 24-month follow-up period (Table 3). At three months, the compromised group had infection rates of 72 (32.72%) versus 25 (11.36%) in the adequate group, while delayed wound healing was observed in 44 (20.00%) and 15 (6.82%) participants, respectively. Although these disparities decreased over time, they remained significant at 24 months, with infections reported in 37 (16.81%) of the compromised group versus five (2.27%) of the adequate group. Delayed wound healing at 24 months was 21 (9.54%) in the compromised group compared to only two (0.91%) in the adequate group. These findings underscore the prolonged impact of nutritional status on postoperative recovery.

**Table 3:** Long-Term Postoperative Outcomes (Infections and Delayed Wound Healing) by Nutritional Status

Follow-up Period (Months)	Outcome	Nutritionally Adequate (n = 220)	Nutritionally Compromised (n = 220)
3 Months	Infections	25 (11.36%)	72 (32.72%)
	Delayed Wound Healing	15 (6.82%)	44 (20.00%)
6 Months	Infections	21 (9.54%)	65 (29.54%)
	Delayed Wound Healing	12 (5.45%)	38 (17.27%)
12 Months	Infections	15 (6.82%)	57 (25.90%)
	Delayed Wound Healing	9 (4.09%)	32 (14.54%)
18 Months	Infections	8 (3.63%)	48 (21.81%)
	Delayed Wound Healing	5 (2.27%)	26 (11.81%)
24 Months	Infections	5 (2.27%)	37 (16.81%)
	Delayed Wound Healing	2 (0.91%)	21 (9.54%)

Nutritionally compromised patients experienced significantly poorer surgical outcomes compared to nutritionally adequate patients (Table 4). They had a longer average hospital stay (9.87 ± 3.58 days vs. 6.53 ± 2.31 days, t = -12.42, p < 0.001) and higher rates of postoperative infections (n=80; 36.36% vs. n=30; 13.64%), χ² = 25.67, p < 0.001), delayed wound healing (n=50; 22.73% vs. n=20; 9.09%), χ² = 10.92, p = 0.001), 30-day postoperative mortality (n=25; 11.36% vs. n=5; 2.27%), χ² = 13.88, p < 0.001), and readmissions (n=40; 18.18% vs. n=10; 4.55%), χ² = 16.48, p < 0.001). These results strongly highlight the negative impact of poor nutritional status on surgical outcomes.

**Table 4:** Association Between Nutritional Status and Surgical Outcomes Data are presented as n (%)

Outcome	Nutritionally Adequate	Nutritionally Compromised	Test	Value	p-value
Length of Hospital Stay (Days)	6.53 ± 2.31	9.87 ± 3.58	t-test	t = -12.42	<0.001
Postoperative Infections	30 (13.64%)	80 (36.36%)	Chi-Square	*X*^2^ = 25.67	<0.001
Delayed Wound Healing	20 (9.09%)	50 (22.73%)	Chi-Square	*X*^2^ = 10.92	0.001
30-Day Postoperative Mortality	5 (2.27%)	25 (11.36%)	Chi-Square	*X*^2^ = 13.88	<0.001
Readmission Rates	10 (4.55%)	40 (18.18%)	Chi-Square	*X*^2^ = 16.48	<0.001

## Discussion

Our research shows that preoperative nutritional status and surgical outcomes are significantly correlated in patients undergoing general surgery, with those who are nutritionally deficient experiencing noticeably lower outcomes after surgery. Compared to their appropriately fed counterparts, patients with nutritional deficiencies had greater rates of surgical complications, infections, delayed wound healing, longer hospital stays, higher readmission rates, and higher 30-day death rates.

Postoperative infection rates were substantially greater in patients who were categorized as nutritionally challenged (36.36% vs. 13.64%, p < 0.001). These results are in line with earlier research that found that malnourished postoperative patients had elevated infection rates because of delayed tissue healing and a compromised immune response [16]. Compared to 9.09% in the group that was appropriately fed, 22.73% of patients with nutritional deficiencies had delayed wound healing (p = 0.001). This is consistent with other studies that found malnourished individuals had delayed wound healing rates of 20-25%, which were linked to shortages in protein and micronutrients necessary for collagen formation [17].

Patients with dietary deficiencies had a considerably longer average hospital stay (9.87 ± 3.58 days vs. 6.53 ± 2.31 days, p < 0.001). Prior research found that malnourished surgery patients have longer hospital stays as a result of greater incidence of complications [18]. Interestingly, our research also revealed that the group with dietary deficiencies had greater readmission rates (18.18% vs. 4.55%, p < 0.001). These findings are consistent with other studies that found a correlation between higher healthcare resource consumption and poor preoperative nutritional status [19].

The 30-day postoperative death rate for nutritionally challenged patients was 11.36%, which was substantially greater than the 2.27% rate for the appropriately fed group (p < 0.001). These death rates are in line with other research that has shown preoperative malnutrition to be a risk factor for higher postoperative mortality on its own [20].

The influence of nutritional status is further shown by long-term outcomes over a 24-month period, which show that infections are still far more common in the impaired group (16.81% vs. 2.27% after 24 months). Previous studies have shown similar long-term patterns, highlighting the long-term impacts of poor nutritional health on recovery paths [21].

Our results provide fresh perspectives on the unique difficulties experienced by patients undergoing general surgery in our context, while also substantially supporting the data that has already been established. Targeted dietary treatments should be investigated in future research to reduce these hazards and enhance results.

Strengths and limitations

The benefits of this research are its large sample size, meticulous methodology, and 24-month follow-up duration, which provide comprehensive insights into how preoperative nutritional state affects surgery outcomes over a long length of time. The accuracy of nutritional evaluations is strengthened by the use of approved instruments, such as blood albumin levels and NRS-2002. However, one limitation is the exclusion of certain nutritional biomarkers, such as prealbumin and transferrin. These markers were not included due to practical considerations, including resource availability and the fact that prior studies have demonstrated serum albumin as a reliable marker for nutritional assessment in surgical patients. The absence of these additional markers may limit the comprehensiveness of the nutritional assessment and the generalizability of the findings.

Additionally, the study's observational design restricts the ability to draw inferences about causality, and the single-center design may limit the results' generalizability. Furthermore, variables that may have affected results, such as variations in surgical complexity and adherence to postoperative care, were not controlled. To overcome these constraints, multicenter research with a more diverse cohort and more comprehensive inclusion of nutritional biomarkers is required in the future.

## Conclusions

According to our research, preoperative nutritional status has a major influence on surgical outcomes for patients undergoing general surgery. Those who are nutritionally compromised have higher rates of infections, delayed wound healing, longer hospital stays, and higher mortality rates following surgery. In order to enhance surgical recovery, our results highlight the vital need of early nutritional evaluation and optimization as part of preoperative treatment. Incorporating nutritional evaluations into standard surgical treatment might improve patient outcomes and reduce the burden on healthcare systems, highlighting the need to treat malnutrition as a critical component of surgical success given the wider public health consequences. To reduce these hazards and enhance patient recovery, future studies should concentrate on creating specialized dietary therapies.
